# Different Outcomes of Experimental Hepatitis E Virus Infection in Diverse Mouse Strains, Wistar Rats, and Rabbits

**DOI:** 10.3390/v11010001

**Published:** 2018-12-20

**Authors:** Josephine Schlosser, Lisa Dähnert, Paul Dremsek, Kerstin Tauscher, Christine Fast, Ute Ziegler, Albrecht Gröner, Rainer G Ulrich, Martin H Groschup, Martin Eiden

**Affiliations:** 1 Institute of Novel and Emerging Infectious Diseases, Friedrich-Loeffler-Institut, 17493 Greifswald-Insel Riems, Germany; josephine.schlosser@gmx.de (J.S.); lisa.daehnert@fli.de (L.D.); p.dremsek@labormustafawien.at (P.D.); Christine.fast@fli.de (C.F.); ute.ziegler@fli.de (U.Z.); Rainer.ulrich@fli.de (R.G.U.); martin.groschup@fli.de (M.H.G.); 2 Department of Experimental Animal Facilities and Biorisk Management, Friedrich-Loeffler-Institut, Südufer 10, 17493 Greifswald-Insel Riems, Germany; kerstin_tauscher@gmx.de; 3 PathoGuard Consult, 64342 Seeheim-Jugenheim, Germany; info@patho-guard.com; 4 German Center for Infection Research (DZIF), partner site Hamburg-Lübeck-Borstel, 17493 GreifswaldInsel Riems, Germany

**Keywords:** Hepatitis E virus, genotype 3, wild boar, mouse, rat, rabbit, knockout mouse, BALB/c, C57BL/6, animal model

## Abstract

Hepatitis E virus (HEV) is the causative agent of acute hepatitis E in humans in developing countries, but autochthonous cases of zoonotic genotype 3 (HEV-3) infection also occur in industrialized countries. In contrast to swine, rats, and rabbits, natural HEV infections in mice have not yet been demonstrated. The pig represents a well-established large animal model for HEV-3 infection, but a suitable small animal model mimicking natural HEV-3 infection is currently missing. Therefore, we experimentally inoculated C57BL/6 mice (wild-type, IFNAR^−/−^, CD4^−/−^, CD8^−/−^) and BALB/c nude (nu/nu) mice, Wistar rats, and European rabbits with a wild boar-derived HEV-3 strain and monitored virus replication and shedding, as well as humoral immune responses. HEV RNA and anti-HEV antibodies were detected in one and two out of eight of the rats and all rabbits inoculated, respectively, but not in any of the mouse strains tested. Remarkably, immunosuppressive dexamethasone treatment of rats did not enhance their susceptibility to HEV infection. In rabbits, immunization with recombinant HEV-3 and ratHEV capsid proteins induced protection against HEV-3 challenge. In conclusion, the rabbit model for HEV-3 infection may serve as a suitable alternative to the non-human primate and swine models, and as an appropriate basis for vaccine evaluation studies.

## 1. Introduction

Hepatitis E virus (HEV) is the causative agent of hepatitis E in humans and taxonomically classified in the family *Hepeviridae*. It is a small, quasi-enveloped virus with a single-stranded RNA genome of positive polarity [1,2,3]. The virus can cause large epidemics, especially in developing countries, where the virus is primarily transmitted via the fecal-oral route through contaminated water due to poor sanitation [4]. However, emerging cases of sporadic and autochthonous hepatitis E also occur in industrialized countries, including the USA, Japan, and European countries [5]. Recently, several hepeviruses were identified in different mammals, such as rodents, bats, camels, carnivores, and moose. Additionally, more distantly related viruses were discovered in chicken and fish [6,7]. A consensus taxonomy has recently been published which divides the family *Hepeviridae* into two genera designated *Orthohepevirus*, including four species named *Orthohepevirus A* to *D*, and the genus *Piscihepevirus* [8]. Within the species *Orthohepevirus A*, human associated strains were grouped, namely genotypes 1 and 2 (HEV-1, HEV-2) that are restricted to humans and the zoonotic genotypes 3, 4 and 7 (HEV-3, HEV-4, HEV-7) that have been detected in domestic pig, wild boar, deer, and camels [7]. HEV-3-related strains were identified in European rabbits (*Oryctolagus cuniculus*) at multiple locations in the USA [9]; China [10]; and Europe, e.g., France [11] or Germany [12,13]. In Europe and Asia, food-borne zoonotic transmissions of HEV have been primarily associated with domestic pigs, wild boar, and deer as one of the main routes of human autochthonous infections [14,15,16]. In addition to the consumption of contaminated raw or undercooked meat, direct contact with pigs has to be considered as a risk factor for HEV infection [17,18]. Initially, HEV was thought to cause only acute courses, but especially HEV-3 strains are now known to be responsible for chronic hepatitis in immunocompromised patients and associated with extrahepatic manifestations [18,19,20].

Pigs, being the natural host species for HEV-3 and HEV-4, can be used as a homologous animal model system for HEV studies [21,22]. Laboratory mice and rats have also been explored as potential animal models for HEV. Although an earlier study indicated the susceptibility of Wistar rats to HEV-1 infection [23], recent trials aiming to infect Wistar rats with HEV-1 or HEV-2 failed [24,25]. Moreover, experiments to infect laboratory rats with HEV-3 were also not successful [24,25,26]. Injection of transcripts of a HEV-4 cDNA into the liver of rats led to a transient seroconversion [27]. This genotype was also shown to be infectious for BALB/c nude mice [28]. Another study in C57BL/6 mice demonstrated that animals intravenously inoculated with HEV-1 to HEV-4 were not susceptible to HEV [29]. Recently, a new liver chimeric mouse model was established in which human hepatocytes that were susceptible to HEV-1 and HEV-3 infection were transplanted [30]. Furthermore, experimental inoculation of rabbits with HEV-3 and HEV-4 strains resulted in seroconversion, but virus shedding and viremia were exclusively assigned to the HEV-4 strain used [30]. Experimental inoculation of rabbits with rabbit HEV led to seroconversion, fecal virus shedding, viremia, and elevated liver enzyme levels [31,32], and could result in chronic infections [33]. Pigs and macaques inoculated with rabbit HEV strains developed transient viremia and virus shedding, thus indicating the zoonotic potential of the virus [34,35].

Currently, experimental studies on pig- and wild boar-derived HEV-3 strains in rodents and rabbits are missing. As recently shown, HEV-3 infection in European wild boar (*Sus scrofa scrofa*) is naturally and experimentally transmissible to domestic pigs (*Sus scrofa domestica*), causing a variable degree of hepatic lesions [36]. As HEV has the ability to cross species barriers, it is important to prove the possibility of cross-species transmission between wild boar and rodents, and lagomorphs, i.e., rabbits. Establishing a small animal model (e.g., in immunodeficient mice) mimicking natural HEV-3 infection would be helpful to gain a better understanding of disease, as this may provide novel mechanistic insights into HEV replication and host immune defense to infection, which is limited in large animal models like the pig. Therefore, we experimentally inoculated C57BL/6 mice (wild-type, IFNAR^−/−^, CD4^−/−^, CD8^−/−^) and BALB/c nude mice, Wistar rats, and rabbits with HEV-3. To determine their susceptibility to HEV infection, viral replication and shedding, as well as the humoral immune response, were monitored. In addition, we also assessed whether dexamethasone treatment has an effect upon the susceptibility of rats to HEV-3 infection. Finally, the protective potential of recombinant HEV vaccine candidates was evaluated exemplarily in rabbits challenged by HEV-3 inoculation.

## 2. Materials and Methods

### 2.1. Inocula

The HEV-3 strain used in this study originated from a liver sample of a naturally infected wild boar hunted in Northern Germany (Mecklenburg-Western Pomerania) in 2009, subtype 3b (accession number: JQ807477.1). The preparation of the liver suspension was performed as described before [36]. The inoculum contained about 2.0 × 10^4^ HEV RNA copies per µL RNA [cop/µL], as shown by RT-qPCR. Additionally, bile (1.1 × 10^4^ cop/µL) and feces (3.1 × 10^3^ cop/µL) of intravenously HEV-3 infected wild boar were used for the inoculation of different mouse strains. The corresponding genome numbers per ml are 7.3 × 10^6^ (liver homogenate, Supplemental Figure S1), 2.9 × 10^6^ (bile), and 1.1 × 10^6^ (feces). In doing so, bile was diluted in phosphate-buffered saline (PBS; 20%, *w*/*v*), sterile-filtered (0.22 µm MILLEX^®^GP filter unit), and aliquoted in volumes of 2.5 mL and stored at −70 °C. Feces was suspended in PBS at a proportion of 20% (*w*/*v*). The fecal suspension was transferred to a 15 mL tube and mixed for 1 min using a vortex mixer. After centrifugation (20 min at 4000× *g* at 4 °C), the supernatant was transferred to a new tube and filtered (0.22 µm MILLEX^®^GP filter unit). The suspension was aliquoted in volumes of 2.5 mL and stored at −70 °C.

### 2.2. Animals and Experimental Design

Wild-type, type I interferon receptor knockout mice (IFNAR^−/−^, B6-129Sv/Ev-IFNabRtm Agt), CD4^−/−^ (B6-CD4tm1 Mak) and CD8^−/−^ (B6-CD8atm1 Mak) mouse strains with the genetic background of C57BL/6 mice (*Mus musculus*) were bred in the specific-pathogen-free (SPF) breeding unit of the Friedrich-Loeffler-Institut, Insel Riems, Germany. The athymic BALB/c nude (nu/nu) mice (*Mus musculus*; CAnN.Cg-Foxn1^nu^/Crl, homozygous) were obtained from Charles River Laboratories, Sulzfeld, Germany. Female Wistar rats (*Rattus norvegicus*; Wistar RccHan™) were purchased from Harlan Laboratories, Venray, The Netherlands. Adult European rabbits were obtained from the quarantine and breeding facility of the Friedrich-Loeffler-Institut, Insel Riems, Germany. The experiments were approved by the competent authority of the Federal State of Mecklenburg-Western Pomerania, Germany, on the basis of national and European legislation, namely the EU council directive 86/609/EEC for the protection of animals used for experiments (LALLF M-V/TSD/7221.3-2.1.-014/10). Prior to the start of the experiments, all animals were shown to be negative for anti-HEV antibodies in serum and/or HEV RNA in feces, respectively. Following an initial clinical examination, all animals were allowed to get accustomed to the new surroundings for approximately one to two weeks prior to the initiation of experiments. All animals, except the BALB/c nu/nu mice, were fed with commercial feed and had access to water ad libitum. The experiments were carried out under biosafety level 3** conditions. The isolator-maintained BALB/c nu/nu mice were kept in an SPF area including autoclaved feed and drinking water.

In general, blood and fecal samples were collected at consecutive time points every two to four days during the time course of the experiment and at necropsy and analysed once with the corresponding method. Additionally, rectal temperatures were measured in Wistar rats and rabbits.

Aliquots of serum samples were stored at −20 °C for antibody detection. Fecal samples were diluted in isotonic saline solution (10%, *w*/*v*) and stored at −70 °C for RNA extraction. Animals were euthanized by exsanguination under anesthesia and samples were collected from their liver, gall bladder (except rats), small and large intestine, kidney, spleen, heart, brain, and *quadriceps femoris* muscle. Aliquots of all tissue samples were also stored at −70 °C for RNA extraction.

### 2.3. Inoculation of Animals with a Wild Boar Derived HEV-3 Strain

#### 2.3.1. Inoculation of Mice

In total, six wild-type (C57Bl/6) mice were inoculated concurrently via the oral and intravenous (i.v.) route, either with HEV positive liver homogenate (*n* = 2), HEV positive feces suspension (*n* = 2), or PBS (*n* = 2, control group). The corresponding numbers were 1.8 × 10^6^ copies in 250 µL liver homogenate and 2.8 × 10^5^ copies in 250 µL feces suspension. According to the same scheme, four CD4^−/−^ and 4 CD8^−/−^ mice were inoculated orally and intravenously with the same volumes of liver homogenate and of feces. Again, two PBS controls for each strain were included. In all three experimental setups, sampling time points were 0, 1, 4, 7, 10, 12, 14, 17, 19, and 21 dpi for feces, and 0, 4, 7, 10, 17, and 21 dpi for serum. At 21 dpi, all animals were necropsied.

IFNAR^−/−^ mice were inoculated either orally (group 1, *n* = 3) or i.v. (group 2, *n* = 3) with liver homogenate (1.8 × 10^6^ copies in 250 µL). For each inoculation group, one PBS control was added. Sampling time points for feces and serum were 0, 3, 7, and 14 dpi regarding the oral group. Sampling (feces and serum) of the intravenous group occurred at 0, 2, 6, and 9 dpi. Necropsy was executed at 7 and 14 dpi (group 1) and 9 dpi (group 2), respectively.

Finally, a total number of 16 BALB/c nude (nu/nu) mice were inoculated i.v., either with 80 µL HEV positive liver homogenate (5.8 × 10^5^ copies), 80 µL feces suspension (8.8 × 10^4^ copies), 80 µL bile (2.3 × 10^5^ copies), or 80 µL PBS. In each case, groups of four animals were inoculated and co-habited with a single untreated mouse, respectively, which served as indicators of contact infection. Due to the small amounts, no RNA extraction was possible from the sampled serum. In general, inoculations were performed either by the injection of material into the lateral tail vein (*Vena coccygea lateralis*) and/or by applying material orally by gavage. Mice receiving sterile-filtered PBS (0.22 µm MILLEX^®^GP filter unit) served as a negative control.

All animals were checked for clinical signs every day during the experiment, including the measurement of the body weight.

#### 2.3.2. Inoculation of Wistar Rats

In the first experimental approach, eight rats were inoculated intravenously into the lateral tail vein (*Vena coccygea lateralis*), receiving 0.25 mL HEV positive liver suspension containing 1.8 × 10^6^ copies and accordingly, eight rats serving as mock controls received 0.25 mL PBS. One additional rat was co-habited with the rats receiving HEV-3-containing liver suspension (total number of animals: 17). Feces was collected at 0, 2, 4, 7, 10, 14, 17, 21, 25, 28, and 32 dpi, and serum at 0, 4, 7, 14, 21, and 32 dpi. At 4, 7, 14, and 21 days post inoculation (dpi), two rats of each intravenously inoculated group were necropsied successively. The co-habited rat was euthanized at 32 dpi.

In a second approach, the potential influence of the immune status on infection dynamics and shedding was examined by pre-treatment of the 12 rats with dexamethasone (0.15 mg/kg, subcutaneously, Voren Suspension^®^, Boehringer Ingelheim Vetmedica, Ingelheim, Germany) at days 7, 4, and 1 prior to inoculation. Eight rats simultaneously received 0.25 mL liver suspension intravenously and orally. Accordingly, four negative control rats received PBS. Sampling of feces was carried out at 0, 2, 4, 7, 14, 17, and 21 dpi, and serum at 0, 4, 7, 14, and 21 dpi. Two intravenously inoculated rats and one control rat, respectively, were subsequently necropsied at 4, 7, 14, and 21 dpi.

All animals were checked for clinical signs every day during the experiment, including the measurement of the body weight. Serum samples were analysed exclusively for serological analysis (ELISA).

#### 2.3.3. Inoculation of European Rabbits

Two independent vaccination experiments were performed in European rabbits according to a standard immunization procedure: Initially, rabbits were immunized subcutaneously with 100 µg antigen and complete Freund’s adjuvant, followed by two consecutive boosts four and eight weeks dpi in incomplete Freund’s adjuvant. Two weeks after the second boost, animals were inoculated correspondingly. In the first experiment (animal number: 3), one rabbit was immunized with an *Escherichia coli* expressed and purified His-tagged C-terminal segment of the ratHEV capsid protein (ratHEV-Ctr) prior to virus challenge [17]. Thereafter, the rabbit was inoculated intravenously into the ear vein (*Vena auricularis*), receiving 1.0 mL liver suspension containing 7.3 × 10^6^ copies of the HEV-3 genome. One non-vaccinated rabbit served as a positive control and was inoculated intravenously, receiving 1.0 mL liver suspension containing HEV-3 and accordingly, one rabbit received 1.0 mL PBS as a negative control. Feces and blood were sampled at consecutive time points (0, 1, 3, 5, 7, 11, 14, 18, 21, 26, 28, 32, 35, 40, 42, and 45 dpi) till necropsy (45 dpi).

In the second experiment (animal number: 4), two rabbits were immunized with an *Escherichia coli* expressed and purified His-tagged C-terminal segment of the HEV-3 capsid protein (GT3-Ctr) prior to virus challenge [17,36]. Four months later, the rabbits were inoculated intravenously, receiving 1.0 mL liver suspension containing HEV-3. One non-vaccinated rabbit receiving 1.0 mL liver suspension and one rabbit receiving 1.0 mL PBS served as positive and negative controls, respectively. Feces and blood samples were collected regularly (0, 1, 3, 5, 7, 11, 14, 19, 21, 25, 28, 31, 34, 39, 42, 45, and 46 dpi) till necropsy (46 dpi).

### 2.4. Anti-HEV Antibody ELISA and Quantitative Real-Time RT-PCR

Sera were tested for the presence of total anti-HEV antibodies with a species independent HEV-Ab ELISA kit (Axiom, Bürstadt, Germany), according to the manufacturer’s instructions. HEV RNA was detected by a novel diagnostic quantitative real-time RT-PCR assay (RT-qPCR) using the CFX96™ Real-Time System (Bio-Rad Laboratories GmbH, Munich, Germany), as described before [37]. Quantification of RNA was carried out by a standard curve using serial dilutions of an HEV standard (see Supplemental Figure S1). Copy number of the standards was calculated by a synthetic calibrator RNA encompassing the RT-qPCR amplicon and a 5’ T-promotor sequence for in vitro transcription [37]. The limit of detection of about 1 cop/µL was reached at Ct values of ~34. As extraction and amplification control, an RT-qPCR amplifying a fragment of the beta-actin mRNA was performed for all samples [38].

## 3. Results

### 3.1. Inoculation Experiments in Different Mouse Strains

In a pilot study, different C57BL/6 mouse strains were tested for susceptibility to HEV-3. Therefore, groups of wild-type (*n* = 6), IFNAR^−/−^ (*n* = 8), CD4^−/−^ (*n* = 6), and CD8^−/−^ (*n* = 6) mice were inoculated intravenously and/or orally by HEV-3 positive liver or feces suspensions or PBS as a negative control. In a second approach with higher animal numbers per group, BALB/c nude mice (*n* = 16) were inoculated intravenously by liver, feces, or bile suspensions containing HEV-3 or PBS as a negative control. There was no evidence of a clinical disease in any of the challenged wild-type or immunodeficient mouse strains. The body weights remained within normal limits. In none of the inoculated mouse strains was HEV RNA detected in feces or tissue samples taken at necropsy. Additionally, the co-habited BALB/c nude mice (*n* = 4) had no clinical signs and did not shed the virus.

### 3.2. Infection Trial in Dexamethasone Treated and Non-Treated Wistar Rats

In a first experiment, eight Wistar rats were challenged with HEV-3 intravenously, while another eight rats were mock controls and one additional rat was a contact exposure control. None of the challenged rats showed clinical symptoms, and their body weights and rectal temperatures remained within normal limits. Anti-HEV antibodies were seen in two out of eight intravenously inoculated rats after 14 (OD_450_: 2.9, cut-off: 0.19) and 21 dpi (OD_450_: 1.6, cut-off: 0.19), and HEV RNA in feces was detectable in another rat at 7 dpi (2.2 copies per µL). No viral RNA was found in the tissue of intravenously inoculated rats at necropsy (32 dpi). The contact animal did not seroconvert within the experiment, but viral RNA was found in the liver (4.4 copies per µL). In a second experiment, eight dexamethasone-treated rats were simultaneously inoculated intravenously and orally to assess the influence of the immune status on infection dynamics and shedding. Four animals served as mock controls. A slight initial body weight decrease was observed in the dexamethasone-treated rats prior to the inoculation, which normalized during the challenge experiment. Neither feces nor tissue samples taken at necropsy tested positive for HEV RNA, and none of the animals showed detectable anti-HEV antibodies in serum. Moreover, negative controls remained seronegative within the experiment and viral RNA was not detected in any of the tissues and feces tested.

### 3.3. Infection and Vaccination Trial in Rabbits

**Figure 1:**
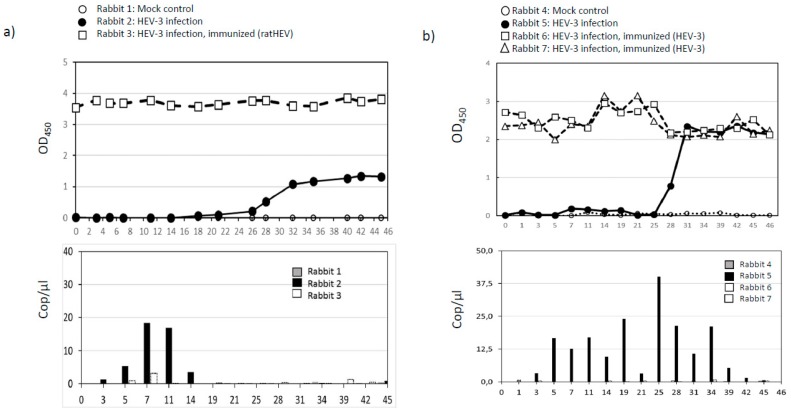
HEV-3 challenge of immunized and naïve European rabbits and induction of protective immunity by immunization with ratHEV capsid protein derivative (**a**) or HEV-3 capsid protein derivative (**b**). (**a**) Immunization with ratHEV capsid protein derivative (ratHEV-Ctr) and subsequent challenge with wild-boar derived HEV-3. [**●**] one non-vaccinated rabbit (Rabbit 2) inoculated with HEV-3; [□] rabbit 3 immunized with a C-terminal segment of ratHEV capsid protein derivative and inoculated with HEV-3 obtained from a wild boar liver; control [○] one rabbit (rabbit 1) inoculated with PBS (negative control). dpi = days post inoculation. (**b**) Immunization with HEV-3 capsid protein derivative (GT3-Ctr) and challenge with HEV-3. [**●**] one non-vaccinated rabbit (rabbit 5) inoculated with HEV-3; [**∆**, □] two rabbits (rabbits 6 and 7) immunized with a C-terminal segment of HEV-3 capsid protein and inoculated with HEV-3 obtained from a wild boar liver; control [○] one rabbit (rabbit 4) inoculated with PBS (negative control).

In the first experiment, one rabbit was inoculated intravenously with liver homogenate containing HEV-3, one rabbit was immunized with a recombinant C-terminal fragment of the ratHEV capsid protein (ratHEV-Ctr) prior to the challenge, and another rabbit served as a negative control. Rabbits showed no clinical signs as the body weights and rectal temperatures remained within normal limits. The intravenously inoculated rabbit (rabbit 2) seroconverted within 28 dpi (Figure 1a). Viral RNA was detected in feces from 3 to 11 dpi, but not in tissue samples taken at necropsy (45 dpi), in contrast to the vaccinated and challenged rabbit (rabbit 3), where viral RNA was shed at 7 and 40 dpi; however, at very low copy numbers of about 3 and 1 cop/µL (Figure 1b). The mock-infected control (rabbit 1) remained negative during the course of the experiment. In the second approach, two rabbits (rabbits 6 and 7) were immunized with a recombinant C-terminal fragment of the HEV-3 capsid protein (GT3-Ctr) prior to the challenge (Figure 1b). The same experimental setup as described before was used for the control animals, i.e., one non-vaccinated rabbit (rabbit 5) was challenged intravenously and one non-vaccinated rabbit (rabbit 4) served as a mock control. Again, none of the animals showed any clinical symptoms. Seroconversion in the intravenously inoculated rabbit which was not vaccinated started after 28 dpi and a slight booster effect was observed in the immunized inoculated rabbits at 14 to 25 dpi. Fecal RNA excretion was detected in the intravenously inoculated non-vaccinated rabbit from 3 to 42 dpi, but not in the immunized rabbits (Figure 1b). Moreover, HEV RNA was found in the liver and gall bladder of the intravenously inoculated non-vaccinated rabbit. In the immunized and challenged rabbits, viral RNA was not detectable in tissue upon necropsy. The mock controls were negative for anti-HEV antibodies or HEV RNA, respectively. In neither case could viral RNA be detected in serum samples.

Antibody responses to HEV in rabbit sera were measured by a double-antigen sandwich ELISA (given as optical density at 450 nm, OD450) and fecal excretion of viral RNA was quantified by RT-qPCR (given as genome copies per µL RNA, cop/µL).

## 4. Discussion

Several types of animal models for HEV infection have been described previously [39,40]. In general, non-human-primates are the most suitable model animals as they can be infected with a variety of HEV genotypes. Domestic pigs have been successfully infected with HEV-3 and HEV-4, and represent a suitable large animal model for HEV infection [36,41,42]. However, primate and swine HEV infection models are quite complex and limited, so a small animal model for infection would be desirable. Thus far, laboratory mice, rats, and European rabbits have been explored as potential animal models for different HEV strains with diverging results [23,24,28,31,32], but it has not been resolved yet whether those species are also susceptible to an HEV-3 strain derived from wild boar.

In this study, an experimental HEV-3 infection of different C57BL/6 mouse strains (wild-type, IFNAR^−/−^, CD4^−/^, CD8^−/−^) and BALB/c nude mice failed to suggest natural resistance to wild boar-derived HEV-3. In accordance with the results described here, Li et al. also failed to infect C57BL/6 mice with domestic pig-derived HEV-3, as well as with HEV-1 and HEV-4 strains [29]. In contrast, a former study showed that male BALB/c nude mice can be infected with an HEV-4 isolate derived from a domestic pig [28]. Unfortunately, the zygosity of the BALB/c nude mice in the previous study remained unclear, as we used homozygous mice. However, the discrepancy in the susceptibility of nude mice to HEV infection might also be due to gender effects (females in our study versus males in the former study).

RatHEV was frequently detected in rats of different species throughout the world [43], whereas HEV-3 was only reported in rats from the USA [44] and Japan [45]. Recently, a liver transplant recipient reported hepatitis caused by ratHEV infection [46]. A homologous challenge of Wistar rats using ratHEV resulted in virus replication and seroconversion [24]. Controversial data have been obtained for the susceptibility of rats to primate- and swine-derived HEV strains. Wistar rats could be experimentally infected with a human HEV strain of an unknown genotype in earlier studies [23]. Contrary, Wistar rats were resistant to intravenous inoculation of HEV-1 originating from a cynomolgus monkey, HEV-3 collected from a domestic pig, and a wild boar-derived HEV-4 strain [24]. In this study, HEV RNA and anti-HEV antibodies were detectable in a small portion of intravenously inoculated Wistar rats, pointing towards susceptibility to HEV-3 of wild boar origin. However, data indicate that Wistar rats might not represent a reliable animal model for HEV-3 infection. Interestingly, dexamethasone treatment in rats did not enhance the susceptibility to HEV infection, but resulted in the complete absence of seroconversion and RNA replication in all inoculated rats. Beside their main effects as immunosuppressants, treatments with glucocorticoids can have also immunostimulatory effects on the immune system. For example, glucocorticoids can prime NK cells for proinflammatory cytokine production [47]) and can also exert opposing effects on macrophage function, depending on their concentration [48]. Probably, similar immunostimulatory effects of dexamethasone might have accelerated virus clearance in our study. An in vitro study showed that dexamethasone treatment could also slow down virus replication of another RNA virus [49]. Hence, a longer observation period (>21 dpi) in our study would have been useful to detect late virus replication. Similarly, another group found no evidence that nude rats are susceptible to infection with HEV-3 [26], but ratHEV showed enhanced viral replication in these rats compared to immunocompetent rats [24].

Here, the experimental HEV-3 infection of European rabbits with a wild boar-derived strain led to seroconversion within four to five weeks post inoculation. Fecal virus shedding and HEV RNA in the liver and gall bladder could also be demonstrated. Our findings regarding the anti-HEV antibody responses are in accordance with another study in rabbits inoculated with HEV-3 [31], but in contrast to our results, viral replication could not be detected in this previous study. A similar study with a human HEV-3 strain again showed seroconversion, but no virus shedding in rabbits [50]. Since human-derived HEV-3 strains were used in these studies, it cannot be excluded that HEV-3 of wild boar origin replicates more efficiently in European rabbits. Moreover, the immunization of rabbits with an HEV-3 recombinant capsid protein derivative produced a robust anti-HEV antibody response and completely protected against HEV-3-challenge, as no HEV RNA was detected in feces and different tissue samples tested. To analyze the antigenic similarity of HEV-3 and ratHEV capsid protein derivatives and to evaluate potential cross protection, immunizations were performed with both antigens. Interestingly, ratHEV-derived capsid protein immunization resulted in a strong antibody response and almost complete protection against HEV-3 challenge. Only at two time points (7 dpi and 40 dpi) were very low virus numbers shed to feces. A booster effect was seen in immunized animals two weeks post inoculation, indicative for antigen-specific memory B cells. In another study, HEV p179 vaccinated rabbits also produced high anti-HEV titers and were completely protected against challenge infection [31]. A more recent study indicated that HEV p239, another HEV vaccine candidate, is highly immunogenic in rabbits and provided complete protection of rabbits against homologous and heterologous HEV challenge [51]. Additionally, rabbits can be experimentally infected with HEV-4 originating from acute hepatitis E patients, but not with a human HEV-1 strain [31,32]. In this regard, it is of interest that HEV sequences of a human strain in France and rabbit strains were closely related, sharing a 93-nucleotide insertion within the ORF1 genomic region [11]. As recently shown, rabbit HEV, but not ratHEV, is able to infect domestic pigs in an experimental approach [34]. Interestingly, a study in China found no evidence of natural cross-species transmission of rabbit HEV between pigs and rabbits [51]. Although rabbit HEV belongs to the same genotype and serotype as human-derived HEV-3 [52,53], the antigenic relationship between rabbit HEV and the wild boar-derived HEV-3 used in this study remains unclear. Remarkably, we could also show that immunization with a recombinant ratHEV capsid protein derivative conveys cross-protection to HEV-3 challenge.

**Figure 2:**
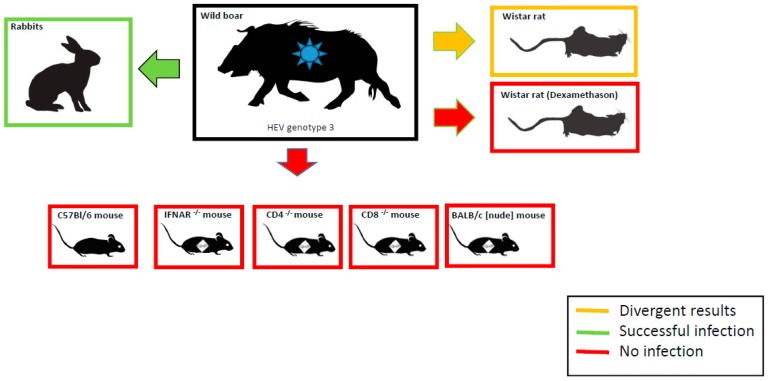
Experimental infection of wild boar-derived HEV-3 strain in wild-type and knockout mouse strains, Wistar rats, and rabbits. Details of the different experiments presented are given in the text.

In conclusion, wild-type and immunodeficient mice are resistant to HEV-3 infection. HEV RNA and anti-HEV antibodies could be detected in rabbits and a portion of the rats inoculated, indicating a productive infection. A summary of experimental HEV infection is depicted in Figure 2. Simultaneous oral and intravenous HEV-3 inoculation did not enhance the susceptibility of dexamethasone-treated Wistar rats. Vaccination of rabbits with recombinant ratHEV and HEV-3 capsid protein derivatives protects against virus shedding after HEV-3 challenge. Contrary to mice and Wistar rats, European rabbits may serve as an alternative model for HEV-3 infection and as an appropriate basis for vaccine evaluation and antiviral drug testing studies.

## Supplementary Materials

Supplementary materials can be found at http://www.mdpi.com/1999-4915/11/1/1/s1.
