# Supplementary material for: Different Outcomes of Experimental Hepatitis E Virus Infection in Diverse Mouse Strains, Wistar Rats, and Rabbits
Source: Viruses. 2018 Dec 20;11(1):1. doi: 10.3390/v11010001 (PMC6356764; doi:10.3390/v11010001)
Supplement: Supplementary file 1 [file viruses-11-00001-s001.pdf]

Supplemental Figure S1. Amplification curve of HEV specific qRT-PCR

a)

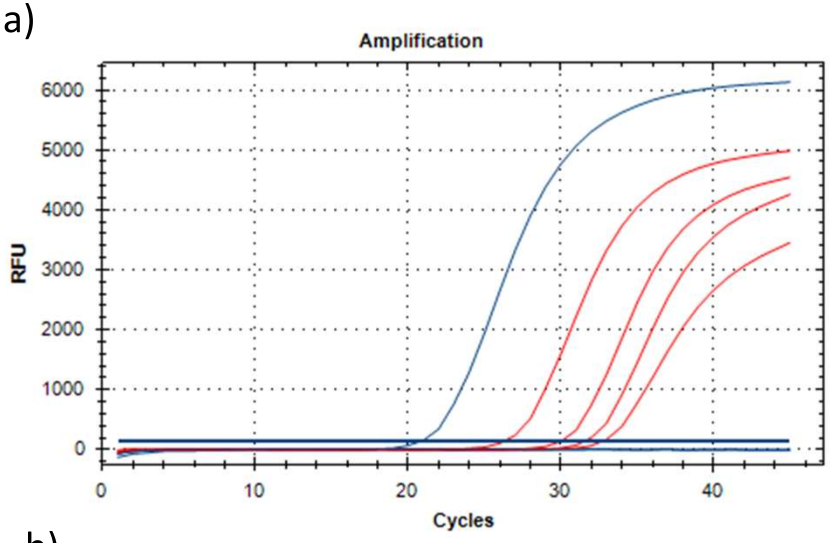

b)

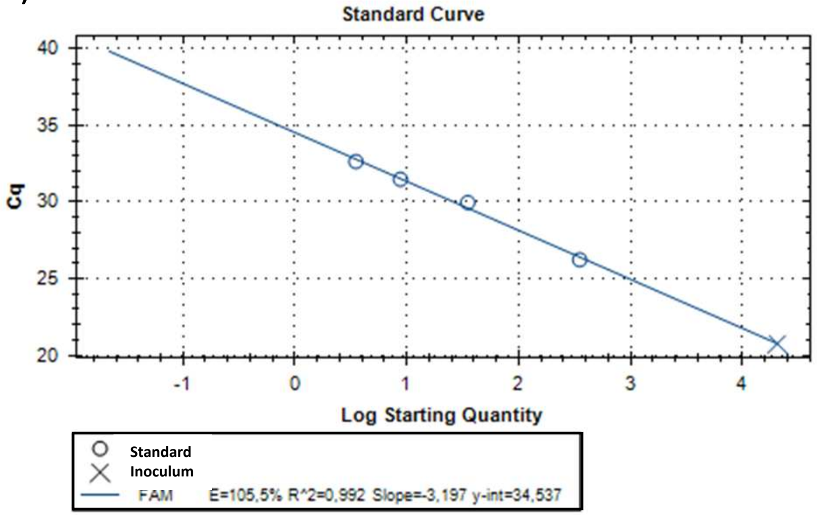

c)

Sample	Ct	Copies per µl RNA	Copies per ml sample*
HEV standard (10^{-2} dilution)	26,23	$3,50x10^{2}$	$1,25x10^{5}$
HEV standard (10^{-3} dilution)	29,97	$3,50x10^{1}$	$1,25x10^{4}$
HEV standard ($10\text{-}^{3/4}$ dilution)	31,49	8,75	$3,12x10^{3}$
HEV standard (10^{-4} dilution)	32,65	3,5	$1,25x10^{3}$
Liver homogenate (inoculum)	20,76	$2,04x10^{4}$	$7,3x10^{6}$
Negative control	N/A	N/A	N/A

Supplemental Figure S1
a) Amplification curve of HEV specific qRT-PCR targeting the HEV standard (red line) and the RNA extracts from the inoculum (blue line).
b) Standard curves were obtained by Ct values plotted against the log starting quantity.
c) Obtained Ct values and determined copy numbers. The concentration [copies/ µl RNA] was obtained from 140µl samples eluted in a 50µl volume. The corresponding conversion factor to calculate copies per ml sample is 357,12
